# Gut microbiota is associated with dietary intake and metabolic markers in healthy individuals

**DOI:** 10.29219/fnr.v66.8580

**Published:** 2022-06-23

**Authors:** Line Gaundal, Mari C. W. Myhrstad, Ida Rud, Terje Gjøvaag, Marte G. Byfuglien, Kjetil Retterstøl, Kirsten B. Holven, Stine M. Ulven, Vibeke H. Telle-Hansen

**Affiliations:** 1 Department of Nursing and Health Promotion, Faculty of Health Sciences, Oslo Metropolitan University, Oslo, Norway; 2 Nofima AS (Norwegian Institute of Food, Fisheries and Aquaculture Research), Ås, Norway; 3 Department of Occupational Therapy, Prosthetics and Orthotics, Oslo Metropolitan University, Oslo, Norway; 4 Mills AS, Oslo, Norway; 5 Department of Nutrition, Institute of Basic Medical Sciences, Faculty of Medicine, University of Oslo, Blindern, Oslo, Norway; 6 The Norwegian National Advisory Unit on Familial Hypercholesterolemia, Department of Endocrinology, Morbid Obesity and Preventive Medicine, Oslo University Hospital, Rikshospitalet, Oslo, Norway

**Keywords:** gut microbiota, metabolic markers, diet, healthy, humans, dietary fiber, vegetables, dietary fat, blood pressure, cholesterol

## Abstract

**Background:**

Metabolic diseases have been related to gut microbiota, and new knowledge indicates that diet impacts host metabolism through the gut microbiota. Identifying specific gut bacteria associated with both diet and metabolic risk markers may be a potential strategy for future dietary disease prevention. However, studies investigating the association between the gut microbiota, diet, and metabolic markers in healthy individuals are scarce.

**Objective:**

We explored the relationship between a panel of gut bacteria, dietary intake, and metabolic and anthropometric markers in healthy adults.

**Design:**

Forty-nine volunteers were included in this cross-sectional study. Measures of glucose, serum triglyceride, total cholesterol, hemoglobin A1c (HbA1c), blood pressure (BP), and body mass index (BMI) were collected after an overnight fast, in addition to fecal samples for gut microbiota analyzes using a targeted approach with a panel of 48 bacterial DNA probes and assessment of dietary intake by a Food Frequency Questionnaire (FFQ). Correlations between gut bacteria, dietary intake, and metabolic and anthropometric markers were assessed by Pearson’s correlation. Gut bacteria varying according to dietary intake and metabolic markers were assessed by a linear regression model and adjusted for age, sex, and BMI.

**Results:**

Of the 48 gut bacteria measured, 24 and 16 bacteria correlated significantly with dietary intake and metabolic and/or anthropometric markers, respectively. Gut bacteria including *Alistipes*, *Lactobacillus* spp., and *Bacteroides stercoris* differed according to the intake of the food components, fiber, sodium, saturated fatty acids, and dietary indices, and metabolic markers (BP and total cholesterol) after adjustments. Notably, *Bacteroides stercoris* correlated positively with the intake of fiber, grain products, and vegetables, and higher *Bacteroides stercoris* abundance was associated with higher adherence to Healthy Nordic Food Index (HNFI) and lower diastolic BP after adjustment.

**Conclusion:**

Our findings highlight the relationship between the gut microbiota, diet, and metabolic markers in healthy individuals. Further investigations are needed to address whether these findings are causally linked and whether targeting these gut bacteria can prevent metabolic diseases.

## Popular scientific summary

- Identifying gut bacteria associated with both diet and metabolic risk markers may be a potential strategy in dietary disease prevention.
- We explored the relationship between a panel of gut bacteria, diet, and metabolic and anthropometric markers in healthy adults.
- *Bacteroides stercoris* was associated with higher intake of healthy foods and lower diastolic blood pressure.
- Further studies are needed to address whether these findings are causally linked and whether targeting these bacteria can prevent metabolic diseases.

New knowledge has pointed out gut microbiota as a mediator of dietary impact on host metabolism (1). The gut microbiota possesses important functions for the host, such as fermentation of dietary fibers and extraction of nutrients, synthesis of certain vitamins, improvement of gut integrity and the protection against pathogens, and the regulation of host immune system and host signaling pathways (2). It can therefore be regarded as a metabolically active organ complementing the host metabolism through its traits (3, 4). In healthy individuals, the gut microbiota comprises a wide range of bacteria belonging to the two major phyla: Firmicutes and Bacteroidetes (5–7). Changes in the gut microbiota composition may disrupt normal functions, and a wide spectrum of diseases, including obesity, type 2 diabetes (T2D), and cardiovascular diseases (CVDs) are associated with gut microbiota dysbiosis (4, 5, 8, 9). Dysbiosis is often referred to as general changes in the gut microbiota composition (10). For example, individuals with lower microbiota gene content are characterized by higher insulin resistance, adiposity, and dyslipidemia than those with higher bacterial richness (11). Furthermore, a promising role of the gut microbiota affecting human metabolism has been shown after microbiota transplant, in which fecal transplantation from lean donors to recipients with metabolic syndrome showed increased gut microbiota diversity and butyrate-producing bacteria, together with improvements in insulin sensitivity (12). The gut microbiota can therefore be an attractive future target for the prevention of metabolic diseases (4).

Both genetic and environmental factors shape the gut microbiota, in which the latter seem to dominate (13–15). Diet is one of the most important factors influencing the gut microbiota composition and function (16–18). The supply of dietary compounds such as non-digestible carbohydrates, protein, and fat strongly impacts the formation of microbiota-derived metabolites (19). A large body of evidence suggest that the beneficial effect of dietary fiber is mediated by the microbial formation of short chain fatty acids (SCFAs), mainly acetate, propionate, and butyrate (20). SCFAs have been shown to regulate metabolic processes, including glucose and lipid metabolism, and are implicated in the regulation of blood pressure (BP) (4, 9, 18, 19, 21). Furthermore, the capability of dietary fats to alter host metabolism through the gut microbiota composition has also been shown (22, 23). Diet-derived free fatty acids may have antimicrobial effects or be used as substrates for the formation of microbiota-derived metabolites, which are involved in inflammatory processes and the regulation of glucose and lipid homeostasis (22, 23). The type of fat also seems to have distinct effects on host metabolism through the gut microbiota. Studies in mice show that dietary fat quality affects inflammation and insulin sensitivity through effects via the gut microbiota composition (24, 25).

Even though studies have shown associations between diet and gut microbiota (26–31), studies investigating metabolic markers associated with both gut microbiota and diet in healthy adults are scarce (32–35). Identifying specific gut bacteria associated with both diet and metabolic markers may be valuable and could represent a potential strategy for the prevention of disease. The aim of this study was therefore to explore the relationship between a panel of gut bacteria, dietary intake, and metabolic and anthropometric markers in a cross-sectional study in healthy individuals.

## Materials and methods

### Subjects and study design

Healthy participants originally recruited to a randomized controlled trial (RCT) (36) were included in this exploratory cross-sectional study. The participants who met to a screening visit prior to the randomized trial performed at Oslo Metropolitan University (OsloMet) were included in this study. Healthy volunteers (aged 18–65 years) with body mass index (BMI) between 18.5 and 27.0 kg/m^2^ were recruited from advertisement and from the student mass and employees at OsloMet between April 2018 and January 2019. Seventy-two volunteers were assessed for eligibility. The exclusion criteria were fasting blood glucose values ≥6.1 mmol/L, any food allergies, intolerances, chronic metabolic diseases (e.g. diabetes, CVD, and cancer), or intestinal diseases, including inflammatory bowel disease (IBD), celiac disease, and irritable bowel syndrom (IBS). Those treated with antibiotics the previous 3 months, who were blood donors the previous 2 months, who were pregnant or lactating, who had ≥5% weight change the previous 3 months, who used any hormonal treatment (except from oral contraception) and tobacco, or who had a high alcohol consume (>40 g/day) were excluded. All participants signed a written informed consent form prior to participation. The study was approved by the Regional Committees for Medical and Health Research Ethics (2018/104) and conducted according to the Declaration of Helsinki guidelines. The study was registered in clinicaltrials.gov (NCT03658681).

### Blood sampling and clinical assessment

Blood sampling and clinical assessment were performed after an overnight fast (≥12 h). Fasting glucose was measured in finger prick capillary blood samples using a HemoCue Glucose 201 Analyser and Micro cuvettes (HemoCue, USA). Fasting triglyceride and total cholesterol were measured in serum and sent to a routine laboratory (Fürst Medical Laboratory) within 24 h. EDTA (Ethylene-Diamine-Tetra-Acetic acid) tubes with whole blood were kept in room temperature for ≤24 h before analyzation of hemoglobin A1c (HbA1c) (Fürst Medical Laboratory). BP was measured twice with an automated BP monitor (ri-champion^©^, Riester, Germany) after sitting still for at least 15 min. Participants were asked to relax during the measurements. The mean value from both measurements was used in the statistical analyses. Body weight and composition were measured after an overnight fast (≥12 h) using Bioimpedance analyzers (BC-418 Segmental Body Composition Analyser and InBody 720). One kg was subtracted compensating for clothing. Height was measured using a wall-mounted stadiometer (Acumed).

### Habitual dietary intake and dietary patterns

Forty-eight of the 49 participants included in this study completed a validated food frequency questionnaire (FFQ), reporting their habitual dietary intake the previous 12 months (37). The FFQ consisted of 270 food items and included questions about frequency of intake (from several times a day to never) and portions size based on household units (slices, glasses, cups, pieces, spoons and teaspoons) (37). Food groups included in the FFQ consisted of categories such as grain products; bread and vegetables; fruits and berries; nuts, olives, and seeds; meat, blood, and offal; fish and shellfish; cheese; and butter, margarine, and oil. Information about the consumption of these food groups and intake of specific foods within these categories (for example, intake of apples within the food group fruits and berries) was used to calculate adherence to dietary indices and to assess correlation with the gut microbiota and metabolic and anthropometric markers.

The dietary indices, the Healthy Nordic Food Index (HNFI) and the Healthy Diet Score (HDS), were used to assess correlation between adherence to the indices and the gut microbiota. The HNFI includes six food groups (fish, cabbage, apple and pears, root vegetables, rye bread, and oatmeal) (38) (Supplementary Table 1). Rye bread was replaced with whole grain bread because information on rye bread specifically was not included in the FFQ, similar to previous studies (39, 40). Thus, in this study, the six food groups included in the modified HNFI were fish (cod, pollack, salmon, trout, herring, mackerel, and shellfish), cabbage (cabbage, cauliflower, broccoli, Chinese cabbage, and Brussels sprouts), apple and pears, root vegetables (carrot and rutabaga), oatmeal, and whole grain bread (>50% whole meal flour). One point was given for intake equal to or above the sex-specific median intake for each food group, and zero points were given for intake below the median. The maximum total score was six points. The total score was summarized for each participant, and a high score (4–6 points) indicates high adherence to the HNFI (38–40).

The HDS reflects adherence to the Norwegian dietary recommendations (41). In this study, the food groups included in the HDS were whole grain, vegetables, fruits and berries, low fat milk, fish, beans and lentils, vegetable oils (monounsaturated fatty acids (MUFAs) and polyunsaturated fatty acids (PUFAs)), red/processed meats, salt, sugar, and saturated fatty acids (SFAs). Each participant was given scores (0, 5, and 10 points) for the intake of food groups according to the Norwegian dietary recommendation and summed into an HDS (Supplementary Table 2). A high score (65–120 points) indicates high adherence to the recommendation, with a maximum score of 120 points.

Findings from a population-based study in Norway show that intakes of dietary fiber, SFA, salt, and sodium are not according to the recommendations, where the intake of fiber is less than recommended and intakes of SFA, sodium, and salt are higher than recommended (42). We therefore assessed whether the intake of these dietary components according to the recommendations was associated with the gut microbiota. Participants were divided into groups based on the average recommendations of fiber set by NNR (Nordic Nutrition Recommendations) (43) (</≥30 g/day, *n* = 19/29). In addition, participants were divided into groups based on recommended intake of SFA (≤/>10 E%/day, *n* = 9/39), salt (≤/>5.0 g/day, *n =* 13/35), and sodium (≤/> 2.3 g/day, *n* = 23/25). Participants were also divided into groups based on high or low adherence to the indices HNFI (0–3/4–6 points, *n* = 30/18) and HDS (0–60/65–120 points, *n* = 10/38).

### Fecal collection and gut microbiota analyses

The participants were provided with a fecal sample collection kit for home use (GA-map™ Dysbiosis Test, Genetic Analysis AS, Oslo, Norway). Participants were instructed to sample the stool from three different places and place it in the included tubes. Samples were kept in room temperature for maximum 3 days according to the manufacturers protocol. The GA-map™ Dysbiosis Test has shown stability in room temperature up to 5 days (44). All samples were stored at −80°C at OsloMet before they were collectively sent to Genetic Analysis AS (GA) for microbiota analyses after this study was completed.

The GA-map™ Dysbiosis Test is a commercially available genome-based test using fecal samples for analyses of gut bacteria associated with dysbiosis, described in detail elsewhere (44). In brief, the test was developed to identify and characterize bacterial groups that were able to distinguish patients with IBS and IBD from healthy controls. The test comprises 48 DNA probes targeting ≥300 bacteria on different taxonomic levels. Probes targeting seven variable regions (V3–V9) of the 16S rRNA gene were used to characterize and identify bacteria present, thus allowing mapping of the intestinal microbiota profile for a selected set of bacteria. Human fecal sample homogenization, and mechanical and enzymatic bacterial cell disruption were utilized to isolate and bind total bacterial genomic DNA to magnetic beads. The hypervariable regions V3–V9 of the 16S rRNA were further amplified by polymerase chain reaction. Single nucleotide extension and hybridization to a complementary DNA strand coupled to beads determined bacterial DNA labeling. To assess the abundance of bacteria, the strength of fluorescent signal (probe intensity) was detected and measured by Luminex 200 (Luminex Corporation). Probes are listed in Supplementary Table 3.

### Statistical analysis

The current cross-sectional study is part of a randomized controlled dietary crossover study, with the primary aim to investigate the effect of fat quality on glycemic regulation, as described previously (36). Thus, this study is an exploratory study investigating the relationship between a panel of gut bacteria, dietary intake, and metabolic and anthropometric markers in healthy individuals. Gut bacteria were log2-transformed before analysis. Correlations between gut bacteria, dietary variables, and metabolic and anthropometric markers were assessed with Pearson’s correlation. Only bacteria showing statistically significant correlations with correlation coefficients ≥0.3 with either dietary intake or anthropometric and/or metabolic markers are presented. Differences in daily intake of dietary nutrients and dietary index score between males and females were assessed by independent sample *t*-test. Participants were divided into groups based on whether the intake was in accordance with the dietary recommendations or not. Differences between log2-transformed gut bacteria based on whether the dietary intake (fiber, sodium, salt, and SFA [E%], and adherence to HNFI and HDS) and metabolic markers (systolic and diastolic BPs and total cholesterol) were below or above the recommended level were thereafter assessed by a linear regression model, hereafter called unadjusted model. Further adjustments for age, sex, and BMI were performed in adjusted models. *P* < 0.05 was regarded as statistically significant. Statistical analyses were performed using IBM SPSS statistic (version 25), and figures were designed using GraphPad Prism 8 for Windows (version 8.0.0.).

## Results

Seventy-two volunteers were assessed for eligibility, and 49 participants (12 males and 37 females) were included in this cross-sectional study. The mean age was 35.6 years (standard deviation [SD] 13.1 years), and measures of BMI, HbA1c, fasting glucose, triglyceride, total cholesterol, and diastolic and systolic BP were within the normal range (Table 1).

**Table 1:** Characteristics of the participants

Anthropometric and biochemical variables		Mean	SD
Female, *n* (%)	37 (75.5)		
Age (years)		35.6	13.1
BMI (kg/m^2^)		22.8	2.2
HbA1c (%)		5.2	0.3
HbA1c (mmol/mol)		33.9	3.4
Glucose (mmol/L)		5.1	0.4
Triglyceride (mmol/L)		0.9	0.4
Total cholesterol (mmol/L)		4.8	0.9
Systolic blood pressure (mmHg)^1^		122.4	15.3
Diastolic blood pressure (mmHg)^1^		71.7	10.7

Variables are measured fasted.

1 Expressed as mean values based on two measurements.

Data on dietary intake were collected by an FFQ reflecting dietary intake the past 12 months (Table 2). Mean daily intake of protein, total fat, MUFA, PUFA, and carbohydrates was 16.0 E%, 36.8 E%, 14.4 E%, 6.7 E%, and 41.4 E%, respectively. The intake of protein, total fat, MUFA, PUFA, and carbohydrates was within the recommended levels, whereas the intake of SFA was higher than recommended (12.5 E%) (45). The intake of fiber, 41.7 and 39.7 g/day for males and females, respectively, was higher than the recommended minimum daily intake, that is, 35 and 25 g/day, respectively (Table 2). Significant gender differences between dietary intake were found only for the intake of starch in g (*P* = 0.005) and E% (*P* = 0.014). For the HNFI and HDS indices, 18 (37.5%) and 38 (79.2%) participants had high adherence to the indices, respectively.

**Table 2:** Daily intake of nutrients and dietary index score assessed by FFQ

Dietary variables	Total (*n* = 48)		Male (*n* = 12)		Female (*n* = 36)	
	Mean g or score^1^ (SD)	Mean E% (SD)	Mean g or score^1^ (SD)	Mean E% (SD)	Mean g or score^1^ (SD)	Mean E% (SD)
kJ	11301.6 (5576.6)		11964.0 (4364.9)		11080.9 (5964.2)	
kcal	2701.2 (1332.8)		2859.5 (1043.2)		2648.4 (1425.5)	
Protein	104.3 (41.7)	16.0 (2.5)	108.7 (36.7)	15.5 (2.3)	102.8 (43.7)	16.2 (2.6)
Alcohol	10.6 (10.0)	2.8 (2.5)	13.2 (12.7)	3.1 (6.1)	9.7 (9.0)	2.8 (2.5)
Total fat	112.6 (67.8)	36.8 (7.3)	113.1 (48.7)	35.3 (2.7)	112.5 (73.7)	37.3 (7.7)
SFA	37.8 (22.7)	12.5 (3.4)	39.9 (24.8)	12.0 (3.8)	37.2 (22.2)	12.6 (3.4)
MUFA	44.4 (29.1)	14.4 (3.3)	42.7 (17.1)	13.5 (2.9)	45.0 (32.3)	14.8 (3.4)
PUFA	20.8 (15.1)	6.7 (2.0)	20.5 (7.6)	6.7 (2.0)	20.9 (16.9)	6.7 (2.0)
Trans-fat	1.0 (0.7)	0.3 (0.2)	1.2 (1.1)	0.4 (0.2)	0.9 (0.6)	0.3 (0.1)
Cholesterol (mg)	310.3 (142.2)		317.3 (174.2)		307.9 (132.9)	
Carbohydrates	278.4 (147.5)	41.4 (7.2)	306.8 (114.1)	43.1 (5.4)	268.9 (157.3)	40.8 (7.7)
Fiber	40.2 (25.8)	2.9 (0.6)	41.7 (18.4)	2.9 (0.7)	39.7 (28.0)	2.9 (0.6)
Starch*	133.0 (61.3)	20.3 (5.3)	170.2 (70.8)	23.9 (5.3)	120.6 (53.3)	19.0 (4.8)
Mono- and disaccharides	126.8 (92.0)	18.3 (6.1)	119.9 (51.6)	16.8 (5.0)	129.1 (102.5)	18.9 (6.4)
Sugar	37.0 (34.1)	5.5 (3.2)	35.1 (22.2)	5.1 (2.7)	37.7 (37.5)	5.6 (3.3)
Salt	6.8 (2.8)		7.0 (3.0)		6.7 (2.8)	
HNFI	3.1 (1.3)		3.2 (1.2)		3.0 (1.3)	
HDS	73.8 (13.9)		70.0 (13.1)		75.0 (14.0)	

1 Mean score of the adapted version of Healthy Nordic Food Index (HNFI) and Healthy Diet Score (HDS). *n* = 48.

* Significant difference in dietary intakes between males and females, assessed by independent sample *t*-test, *P* < 0.05.

**Fig. 1:**
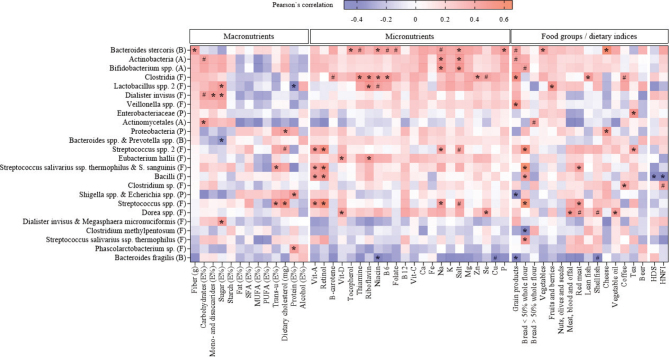
Heat map of Pearson’s coefficient between gut bacteria and dietary intake of macronutrients, micronutrients, and food groups. The bacterial taxa are sorted from negative (blue) to positive (red) correlations in relation to the intake of fiber (g) assessed by Pearson’s correlation. Significant correlations are marked by * (correlation coefficient ≥ 0.3) or # (correlation coefficient < 0.3). Phylum is indicated within parentheses; A, Actinobacteria; B, Bacteroidetes; F, Firmicutes; P, Proteobacteria; Ca, calcium; Cu, copper; E%, percentage of total energy intake; Fe, iron; HDS, Healthy Diet Score; HNFI, Healthy Nordic Food Index; K, potassium; Mg, magnesium; MUFA, monounsaturated fatty acids; Na, sodium; P, phosphorus; PUFA, polyunsaturated fatty acids; Se, selenium; SFA, saturated fatty acids; Vit, vitamin; Zn, zinc.

The abundance of the 48 gut bacteria probes included in this study represents bacteria belonging to the phyla Actinobacteria, Bacteroidetes, Firmicutes, Proteobacteria, Tenericutes, and Verrucomicrobia (Supplementary Table 3). Twenty-four bacteria significantly correlated (correlation coefficient ≥ 0.3) to one or more dietary variables as assessed with Pearson’s correlation. These bacteria and their correlations to dietary variables are illustrated in Fig. 1. Eleven bacteria were related to one or more macronutrients, 12 bacteria were related to one or more micronutrients, and 16 bacteria were related to one or more food groups or dietary indices (Fig. 1).

As shown in Fig. 1, the bacteria correlating with the most dietary variables were *Bacteroides stercoris*, *Clostridia*, and *Streptococcus* spp., showing correlations with 12, 10, and eight dietary variables, respectively. The strongest positive correlation was between *Streptococcus* spp. and the intake of bread with <50% whole flour (*r*: 0.655, *P* < 0.001), whereas the strongest negative correlation was between *Bacteroides* spp. & *Prevotella* spp. and the intake of sugar (E%) (*r*: −0.488, *P* < 0.001). The intake of fiber and fiber-rich foods such as grain products and vegetables, and micronutrients commonly found in these food groups (tocopherol, thiamine, niacin, vitamin B6, folate, and phosphorus) correlated positively with *Bacteroides stercoris* (Fig. 1).

**Fig. 2:**
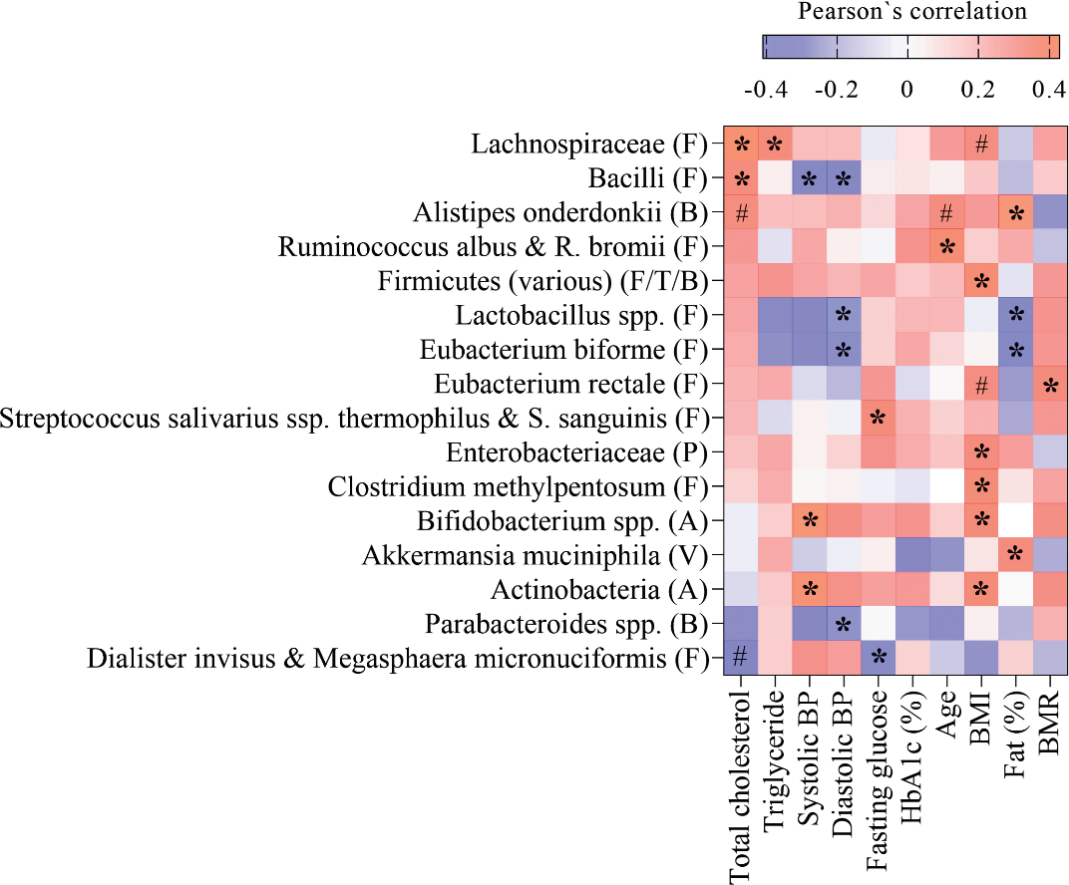
Heat map of Parsons’s coefficient between gut bacterial taxa and metabolic and anthropometric markers. The bacterial taxa are sorted from negative (blue) to positive (red) correlation toward total cholesterol levels, assessed by Pearson’s correlation. Significant correlations are marked by * (correlation coefficient ≥ 0.3) or # (correlation coefficient < 0.3). Phylum is indicated within parentheses; A, Actinobacteria; B, Bacteroidetes; F, Firmicutes; P, Proteobacteria; T, Tenericutes; V, Verrucomicrobia; BMI, body mass index; BMR, basal metabolic rate; BP, blood pressure; HbA1c, hemoglobin A1c.

Correlations between gut bacteria and metabolic and anthropometric markers were thereafter investigated. Sixteen gut bacteria showed a statistically significant correlation with metabolic and/or anthropometric markers (correlation coefficients ≥ 0.3), as outlined in Fig. 2. Of these, two bacteria from the Bacteroidetes phylum (*Alistipes onderdonkii* and *Parabacteroides* spp.) and nine bacteria from the Firmicutes phylum (*Lachnospiraceae*, *Bacilli*, *Ruminococcus albus* & *R. bromii*, *Lactobacillus* spp., *Eubacterium biforme*, *Eubacterium rectale*, *Streptococcus salivarius* spp. *thermophilus* & *S. sanguinis*, and *Dialister invisus* & *Megasphaera micronuciformis*) correlated significantly to metabolic and/or anthropometric markers. In addition, Actinobacteria and *Bifidobacterium* spp. belonging to the Actinobacteria phylum, and *Akkermansia muciniphila* and *Enterobacteriaceae* from the phyla Verrucomicrobia and Proteobacteria, respectively, correlated to metabolic and/or anthropometric markers. The strongest positive correlation was between *Alistipes onderdonkii* and body fat (%) (*r*: 0.428, *P* = 0.002), and the strongest negative correlation was between *Lactobacillus* spp. and diastolic BP (*r*: −0.411, *P* = 0.003). Furthermore, BP correlated with several gut bacteria showing a positive correlation with Actinobacteria and *Bifidobacterium* spp. and a negative correlation with *Parabacteroides* spp., *Bacilli, Eubacterium biforme*, and *Lactobacillus* spp. The abundance of *Lachnospiraceae*, *Bacilli*, *Alistipes onderdonkii*, *Dialister invisus*, and *Megasphaera micronuciformis* were significantly correlated to blood lipids (total cholesterol and/or triglyceride) (Fig. 2).

**Fig. 3:**
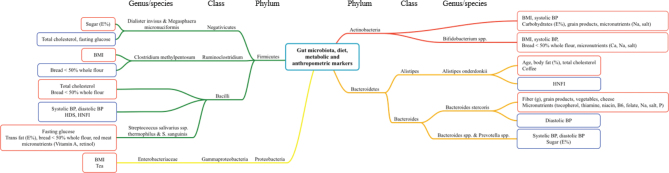
Correlation map between gut bacteria, dietary intake, and metabolic and anthropometric markers. The gut bacteria are grouped according to representative phyla, indicated by different colors (red: Actinobacteria; orange: Bacteroidetes; green: Firmicutes; yellow: Proteobacteria). Correlation between the gut bacteria with one or more dietary variables, metabolic and/or anthropometric markers is illustrated by red boxes (positive correlation) or blue boxes (negative correlation). BMI, body mass index; BMR, basal metabolic rate; BP, blood pressure; E%, percentage of total energy intake; HDS, healthy diet score; HNFI, Healthy Nordic food index.

Based on the correlation analysis shown in Figs. 1 and 2, significant correlations between gut bacteria and both dietary variables and metabolic and anthropometric markers are summarized and illustrated in a correlation map in Fig. 3. These gut bacteria are color coded according to their representative phyla: Actinobacteria, Firmicutes, Bacteroidetes, and Proteobacteria. The abundance of *Bacteroides stercoris* correlated positively with the intake of healthy food groups or components such as fiber, grain products, and vegetables, and negatively with diastolic BP. Furthermore, the abundance of *Bacilli* correlated positively with total cholesterol and the intake of bread with <50% whole flour, and negatively with systolic and diastolic BP and the indices HDS and HNFI. The abundance of *Alistipes onderdonkii* correlated positively with total cholesterol, age, and body fat (%), and negatively with adherence to HNFI (Fig. 3).

As shown in Fig. 3, *Bifidobacterium* spp. and *Streptococcus salivarius* spp. *thermophilus* showed only positive correlations with metabolic and/or anthropometric markers and dietary components (systolic BP, BMI, intake of bread with <50% whole flour, and micronutrients [Ca, Na, and salt]; and fasting glucose, intake of trans fat [E%], intake of bread with <50% whole flour, red meat, and micronutrients [vitamin A and retinol], respectively). *Bacteroides* spp. and *Prevotella* spp. showed only negative correlations with metabolic markers and dietary components (systolic and diastolic BP and intake of sugar [E%], respectively) (Fig. 3).

To investigate if the abundance of gut bacteria differed in relation to the intake of food components, especially linked to health effects, participants were divided into groups based on whether the intake was below or above the recommended intake. Participants were divided based on the average recommended intake of fiber, irrespective of gender (</≥30 g/day) (*n* = 19/29), and the recommended intake of sodium (≤/> 2.3 g/day) (*n* = 23/25), salt (≤/> 5.0 g/day) (*n =* 13/35), and SFA (≤/>10 E%/day) (*n* = 9/39), in addition to high or low adherence to HNFI and HDS indices (0–3/4–6 points (*n* = 30/18), and 0–60/65–120 points (*n* = 10/38), respectively). Gut bacteria showing a significant difference in abundance between groups in the linear regression analyses are shown in Table 3.

**Table 3:** Gut bacteria associated with the intake of fiber, sodium, salt, SFA (E%), and adherence to HNFI and HDS indices^1^

Gut bacteria	Unadjusted values		Adjusted values		*P* ^ǂ^	*P* ^҂^
	*B*	95% CI	*B*	95% CI		
	**Fiber intake (≥ 30 g/d) (*n* = 29)**					
(B) *Alistipes*	−0.306	−1.697, −0.068	−0.301	−1.730, −0.008	**0.034**	**0.048**
	**Sodium intake (> 2.3 g/d) (*n* = 25)**					
(B) *Bacteroides fragilis*	−0.297	−2.672, −0.063	−0.250	−2.495, 0.198	**0.040**	0.093
(B) *Bacteroides stercoris*	0.367	0.325, 2.303	0.329	0.118, 2.241	**0.010**	**0.030**
	**Salt intake (> 5.0 g/d) (*n* = 35)**					
(P) *Proteobacteria*	0.314	0.087, 1.595	0.288	−0.044, 1.585	**0.030**	0.063
	**SFA intake (≥ 10 E%/d) (*n* = 20)**					
(A) *Actinomycetales*	−0.385	−1.928, −0.325	−0.404	−2.079, −0.283	**0.007**	**0.011**
(F) *Firmicutes*	−0.300	−0.766, −0.022	−0.319	−0.839, 0.001	**0.038**	**0.050**
(F) *Ruminococcus gnavus*	0.204	−0.264. 1.520	0.238	0.050, 1.966	0.163	**0.040**
(F) *Phascolarctobacterium* sp.	0.246	−0.202, 2.620	0.331	0.056, 3.187	0.091	**0.043**
(T) *Mycoplasma hominis*	−0.221	−2.175, 0.289	−0.327	−2.716, −0.076	0.130	**0.040**
	**High adherence to HNFI (≥ 4 points) (*n* = 18)**					
(B) *Bacteroides stercoris*	0.333	0.198, 2.266	0.317	0.114, 2.229	**0.021**	**0.031**
(F) *Bacilli*	−0.338	−1.056, −0.182	−0.420	−1.123, −0.219	**0.006**	**0.005**
(F) Lactobacillus spp.	−0.260	−2.481, 0.122	−0.332	−2.721, −0.291	**0.075**	**0.016**
(F) *Eubacterium biforme*	−0.276	−2.639, 0.041	−0.345	−2.905, −0.335	0.057	**0.015**
(F) *Streptococcus salivarius* spp. *thermophilus*	−0.294	−1.556, −0.029	−0.329	−1.673, −0.098	**0.042**	**0.028**
	**High adherence to HDS (≥ 65 points) (*n* = 38)**					
(F) *Lactobacillus* spp. 2	−0.383	−1.680, −0.278	−0.337	−1.618, −0.107	**0.007**	**0.026**

1 Gut bacteria values were log2-transformed before analysis.

ǂ *P* for unadjusted values assessed by a linear regression model.

҂ *P* for values adjusted for age, sex, and BMI, assessed by a linear regression model.

Phyla are indicated within parentheses; (A), Actinobacteria; (B), Bacteroidetes; (F), Firmicutes; (P), Proteobacteria; (T), Tenericutes

The level of significance was set at *P* < 0.05 and are indicated in bold italic.

The intake of fiber (≥30 g/day) was significantly associated with lower abundance of *Alistipes*, whereas higher intake of sodium (>2.3 g/day) was significantly associated with higher abundance of *Bacteroides stercoris* after adjustment for age, sex, and BMI (Table 3). Furthermore, the intake of SFA ≥10 E%/day was significantly associated with increased abundance of *Ruminococcus gnavus* and *Phascolarctobacterium* sp., and lower abundance of *Actinomycetales* and *Mycoplasma hominis* after adjusting for age, sex, and BMI (Table 3). The indices HNFI and HDS were further examined. A high adherence to HNFI (≥4 points) was significantly associated with higher abundance of *Bacteroides stercoris* and lower abundance of *Bacilli*, *Lactobacillus* spp., *Eubacterium biforme*, and *Streptococcus salivarius* spp. *thermophilus*, while a higher adherence to the HDS (≥65 points) was associated with lower abundance of *Lactobacillus* spp. 2 after adjusting for age, sex, and BMI (Table 3).

Differences in the abundance of gut bacteria between participants stratified according to their systolic BP (</≥120 mmHg) (n = 25/24), diastolic BP (</≥80 mmHg) (n = 39/10), and total cholesterol levels (</≥5.0 mmol/L) (n = 29/20) were thereafter investigated. Gut bacteria showing a significant difference in abundance between groups in the linear regression analyses are shown in Table 4. A higher systolic BP (≥120 mmHg) was associated with lower abundance of *Lactobacillus* spp. after adjusting for age, sex, and BMI (Table 4). A higher diastolic BP (≥80 mmHg) was associated with lower abundance of *Bacteroides stercoris*, *Bacteroides* spp., *Bacilli*, *Eubacterium biforme*, *Eubacterium rectale*, *Lactobacillus* spp., and *Streptococcus* spp. 2, and higher abundance of *Dialister invisus* and *Megasphaera micronuciformis*, after adjusting for age, sex, and BMI. A higher total cholesterol level (≥5.0 mmol/L) was associated with higher abundance of *Ruminococcus albus* and *Ruminococcus bromii*, but this association was no longer significant after adjusting for age, sex, and BMI (Table 4).

**Table 4:** Gut bacteria associated with metabolic markers^1^

Gut bacteria	Unadjusted values		Adjusted values		*P* ^ǂ^	*P* ^҂^
	*B*	95% CI	*B*	95% CI		
	**Systolic BP (≥ 120 mmHg) (*n* = 24)**					
(A) *Actinobacteria*	0.285	0.014, 2.028	0.238	−0.196, 1.903	**0.047**	0.108
(A) *Bifidobacterium*	0.305	0.097, 2.201	0.251	−0.152, 2.045	**0.033**	0.090
(F) *Lactobacillus* spp.	−0.229	−2.269, 0.251	−0.309	−2.583, −0.140	0.114	**0.030**
	**Diastolic BP (≥ 80 mmHg) (*n* = 10)**					
(B) *Bacteroides stercoris*	−0.300	−2.570, −0.087	−0.320	−2.718, −0.123	**0.036**	**0.033**
(B) *Bacteroides* spp. and *Prevotella* spp.	−0.288	−1.259, −0.015	−0.225	−1.163, 0.166	**0.045**	0.138
(B) *Bacteroides* spp.	−0.279	−2.348, 0.010	−0.335	−2.635, −0.166	0.052	**0.027**
(F) *Bacilli*	−0.350	−1.286, −0.155	−0.387	−1.404, −0.189	**0.014**	**0.011**
(F) *Dialister invisus* and *Megasphaera*	0.330	0.363, 4.138	0.384	0.644, 4.584	**0.020**	**0.010**
*micronuciformis*						
(F) *Eubacterium biforme*	−0.345	−3.487, −0.389	−0.313	−3.361, −0.157	**0.015**	**0.032**
(F) *Eubacterium rectale*	−0.325	−2.661, −0.209	−0.357	−2.814, −0.343	**0.023**	**0.013**
(F) *Lactobacillus* spp.	−0.378	−3.553, −0.580	−0.348	−3.400, −0.407	**0.007**	**0.014**
(F) *Streptococcus* spp. 2	−0.294	−1.217, −0.029	−0.305	−1.284, −0.007	**0.040**	**0.048**
	**Total cholesterol (≥ 5.0 mmol/L) (*n* = 20)**					
(F) *Ruminococcus albus* and *R. bromii*	0.374	0.433, 2.739	0.245	−0.579, 2.659	**0.008**	0.202

1 Gut bacteria values were log-transformed before analysis.

ǂ *P* for unadjusted values assessed by a linear regression model.

҂ *P* for values adjusted for age, sex, and BMI, assessed by a linear regression model.

Phyla are indicated within parentheses; A, Actinobacteria; B, Bacteroidetes; F, Firmicutes.

The level of significance was set at *P* < 0.05 and are indicated in bold italic.

## Discussion

In this study, we explored the relationship between a panel of gut bacteria commonly found in the human gut, dietary intake, and metabolic and anthropometric markers in healthy adults. Of the 48 gut bacteria analyzed in this study, 24 bacteria were shown to correlate with dietary intake and 16 bacteria correlated with metabolic and/or anthropometric markers. Several of the gut bacteria correlated with both dietary intake and metabolic and/or anthropometric markers. Furthermore, we show that specific gut bacteria differed in relation to the intake of food components such as fiber, sodium, SFA, and healthy food indices, and between participants stratified according to BP and total cholesterol.

The abundance of *Bacteroides stercoris* was positively associated with higher adherence to the HNFI index and negatively associated with a higher diastolic BP after adjusting for age, sex, and BMI. Moreover, *Bacteroides stercoris* correlated positively with the intake of healthy foods and food components, including fiber, grain products, and vegetables. Dietary carbohydrates provide important substrates for microbial metabolism in the human gut. Many members of the *Bacteroides* genus are enriched with genes encoding carbohydrate-active enzymes and are involved in the breakdown of complex carbohydrates from the diet (46–48). As such, *Bacteroides* members are the predominant organisms involved in carbohydrate metabolism and are considered generalists due to their capacity to switch between host and diet-derived energy sources (19). Furthermore, *Bacteroides stercoris* has been identified as part of a common set of microbial species referred to as a common bacterial core, which are largely shared between individuals (7). Interestingly, *Bacteroides stercoris* has been postulated as a keystone species of the human gut microbiome influencing the microbial community structure including the growth of butyrate-producing bacteria (49).

Higher abundance of *Bacteroides stercoris* in healthy individuals compared with patients with IBD has been shown (50), suggesting a potential beneficial impact of the presence of *Bacteroides stercoris* (51). Taken together, these findings indicate a potential protective role of *Bacteroides stercoris* on human health. However, these studies cannot establish causality and the role of *Bacteroides stercoris* for health and disease needs to be further elucidated in intervention studies.

A higher intake of fiber was associated with lower abundance of *Alistipes* after adjusting for age, sex, and BMI in this study. Interestingly, diets low in fiber and high in fats have shown to increase the abundance of *Alistipes* (52, 53). Whether these differences are related to metabolic regulation by diet needs further investigation, as the impact of *Alistipes* has been reported as both protective and detrimental on CVD (54). In addition to *Alistipes*, members of the Proteobacteria phylum are reported to increase with the presence of fat, indicating that these members utilize dietary fats for growth (52). Here, we show that Proteobacteria abundance correlated positively with the intake of dietary cholesterol and cheese. A high-cholesterol diet was recently shown to increase Proteobacteria abundance in zebrafish (55), and high-fat feeding resulted in higher abundance of Proteobacteria in mice (56–58). Furthermore, Proteobacteria has been reported to increase in low-grade inflammation, a common feature of metabolic diseases (59). *Enterobacteriaceae*, a member of the Proteobacteria phylum, correlated positively with BMI in this study. In line with our results, *Enterobacteriaceae* has been associated with obesity (60), while weight-loss has been shown to reduce the abundance of *Enterobacteriaceae* (61–63). Taken together, these findings may indicate that dietary components such as fiber and fat modulate the gut microbiota composition, which in turn may impact host metabolic regulation.

In addition to *Bacteroides stercoris*, we show that a lower abundance of bacteria belonging to the Firmicutes phylum, specifically *Bacilli*, *Eubacterium biforme*, *Eubacterium rectale*, *Streptococcus* spp. 2, and *Lactobacillus* spp., was associated with higher diastolic BP after adjusting for age, sex, and BMI. Moreover, a higher systolic BP was also associated with lower abundance of *Lactobacillus* spp. after the same adjustments. The relationship between *Lactobacillus* abundance and BP has also been reported by others. Palmu and colleagues recently demonstrated a strong negative correlation between certain *Lactobacillus* species and BP, and sodium intake in a Finish cohort including 6,953 participants (64). In a study by Wilck et al., a high salt diet was shown to increase BP and reduce the abundance of *Lactobacillus* species in mice (65). In this study, the administration of *Lactobacillus* species to mice resulted in a reduced systolic BP and normalization of diastolic BP. The authors further tested this effect in a pilot study in healthy males receiving 6 g sodium for 14 days, which led to a higher BP and a loss of *Lactobacillus* species (65). The relationship between *Lactobacillus* and BP was also evaluated in a meta-analysis of RCTs including 702 individuals (66). The effect of probiotic fermented milk, commonly adding *Lactobacillus* species for the fermentation purpose, reduced systolic and diastolic BP with 3.10 and 1.09 mmHg, respectively, compared with placebo, and the effect on systolic BP was suggested to be even more effective in hypertensive individuals (66). Another meta-analysis of RCTs with 543 participants also showed an effect of probiotic consumption on BP (67). The intake of probiotics, including probiotics from dairy, reduced systolic BP with 3.56 mmHg and diastolic BP with 2.38 mmHg compared with control groups. Interestingly, this effect was found to be similar to the effect of salt reduction and resistance training (67). Furthermore, pre-hypertensive and hypertensive individuals displayed a reduced gut microbiota diversity and richness compared with healthy controls (68). Furthermore, transplantation of fecal bacteria from the hypertensive donors to germ-free mice increased BP and reduced microbiota diversity in hypertensive mice compared with control mice (68). These findings may, therefore, indicate that the gut microbiota, in particular the abundance of *Lactobacillus*, may be involved in the regulation of BP.

A positive correlation between *Lachnospiraceae* and fasting total cholesterol and triglyceride levels was found in this study. The gut microbiota has been reported to play an important role in the variation of blood lipid levels in humans (69). A positive correlation between cholesterol levels and *Lachnospiraceae* has been shown previously (70), while others show an inverse relationship (34, 71, 72). *Lachnospiraceae* is a heterogenic family comprised of taxa reported as both potentially beneficial and harmful (73, 74). For example, differences in *Lachnospiraceae* members between healthy controls and patients with T2D and metabolic syndrome have been shown (75, 76), and different members have been related to either higher or lower lifetime CVD risk (77). The heterogeneity of *Lachnospiraceae* emphasizes these discrepancies, and investigating gut microbes in lower taxonomic levels may give more insight to the potential role of *Lachnospiraceae* in host lipid metabolism.

In this study, we demonstrate associations between a panel of gut bacteria, dietary intake, and metabolic and anthropometric markers in healthy adults. This study is explorative by nature, and the results obtained may be used to further generate new hypothesis. Hence, we did not control for multiple testing. In this study, the sample size, the inclusion of relatively young adults (mean age 35.6 years), and mostly women (75.5%) in a Norwegian population residing in the Oslo area represent limitations. In addition, dietary data were collected by a FFQ and showed a high fiber intake (41.7 g/day for males and 39.7 g/day for females). In comparison, findings from the Norwegian Women and cancer cohort, also using FFQ data, reported fiber intakes of 20 g/day (78). Hence, our findings may not be generalized to other populations in different geographical regions as the gut microbiota has been shown to differ among age, gender, and geographical regions (79–81). The findings in this study may, however, highlight the relationship between a high fiber intake and the gut microbiota.

Using a targeted method to detect only 48 bacteria may be criticized, as other bacteria than those analyzed may have an impact on metabolic regulation. However, the method provides a rapid, high-throughput analysis of human fecal samples allowing researchers to assess fecal microbiota composition without advanced expertise. Here, we explored a panel of bacteria commonly found in the human gut, shown to differ between healthy controls and patients with IBD and IBS, characterized as dysbiotic (44). Although no clear consensus about the definition of dysbiosis in the literature currently exist, dysbiosis often refers to as general changes in the gut microbiota composition, an imbalance in composition, or as changes in specific taxa in that composition (10). Here, we further investigate the associations of the selected set of gut bacteria related to dysbiosis with diet and metabolic markers in healthy adults. Identifying potentially harmful or beneficial bacteria by targeted methods may, in the future, be useful to predict disease risk and/or be used as a therapeutic potential for treating disorders. However, the lack of measures of microbial metabolites such as SCFAs limits our possibility to investigate the functionality of the gut microbiota and its relationship with dietary intake and metabolic and anthropometric markers. A complete characterization of the gut microbiota and measures of microbial metabolites are encouraged in future studies. Our findings may provide useful information about the association between a panel of gut bacteria, dietary intake, and metabolic and anthropometric markers in healthy adults. The cross-sectional design does not allow us to establish causality, and we encourage more and larger randomized controlled studies to further investigate this relationship.

## Conclusion

In this study, we demonstrate associations between a panel of gut bacteria, dietary intake, and metabolic and anthropometric markers in healthy individuals. Of specially interest was the abundance of *Bacteroides stercoris*, which correlated positively with the intake of fiber. Further adjustments for age, sex, and BMI showed that *Bacteroides stercoris* was positively associated with higher adherence to the HNFI index and negatively associated with higher diastolic BP. These findings may indicate a role for the gut microbiota in metabolic regulation through diet, or that the gut microbiota may reflect a healthy versus unhealthy lifestyle. Whether we can modify the gut microbiota by diet and consequently impact metabolic status remains to be elucidated.

Data described in the manuscript will be made available upon request pending application and approval.

## Supplementary Material

Gut microbiota is associated with dietary intake and metabolic markers in healthy individuals

## References

[1] Sonnenburg JL, Backhed F. Diet-microbiota interactions as moderators of human metabolism. Nature 2016; 535: 56–64. doi: 10.1038/nature18846 27383980 PMC5991619

[2] Thursby E, Juge N. Introduction to the human gut microbiota. Biochem J 2017; 474: 1823–36. doi: 10.1042/BCJ20160510 28512250 PMC5433529

[3] O’Hara AM, Shanahan F. The gut flora as a forgotten organ. EMBO Rep 2006; 7: 688–93. doi: 10.1038/sj.embor.7400731 16819463 PMC1500832

[4] Koh A, Bäckhed F. From association to causality: the role of the gut microbiota and its functional products on host metabolism. Mol Cell 2020; 78: 584–96. doi: 10.1016/j.molcel.2020.03.005 32234490

[5] Arora T, Backhed F. The gut microbiota and metabolic disease: current understanding and future perspectives. J Intern Med 2016; 280: 339–49. doi: 10.1111/joim.12508 27071815

[6] Eckburg PB, Bik EM, Bernstein CN, Purdom E, Dethlefsen L, Sargent M, et al. Diversity of the human intestinal microbial flora. Science (New York, N.Y.) 2005; 308: 1635–8. doi: 10.1126/science.1110591 PMC1395357 15831718

[7] Qin J, Li R, Raes J, Arumugam M, Burgdorf KS, Manichanh C, et al. A human gut microbial gene catalogue established by metagenomic sequencing. Nature 2010; 464: 59–65. doi: 10.1038/nature08821 20203603 PMC3779803

[8] Hansen TH, Gøbel RJ, Hansen T, Pedersen O. The gut microbiome in cardio-metabolic health. Genome Med 2015; 7: 33. doi: 10.1186/s13073-015-0157-z 25825594 PMC4378584

[9] Hur KY, Lee M-S. Gut microbiota and metabolic disorders. Diabetes Metab J 2015; 39: 198–203. doi: 10.4093/dmj.2015.39.3.198 26124989 PMC4483604

[10] Hooks KB, O’Malley MA, Davies JE. Dysbiosis and its discontents. mBio 2017; 8: e01492-17. doi: 10.1128/mBio.01492-17 29018121 PMC5635691

[11] Le Chatelier E, Nielsen T, Qin J, Prifti E, Hildebrand F, Falony G, et al. Richness of human gut microbiome correlates with metabolic markers. Nature 2013; 500: 541–6. doi: 10.1038/nature12506 23985870

[12] Vrieze A, Van Nood E, Holleman F, Salojarvi J, Kootte RS, Bartelsman JF, et al. Transfer of intestinal microbiota from lean donors increases insulin sensitivity in individuals with metabolic syndrome. Gastroenterology 2012; 143: 913–6.e7. doi: 10.1053/j.gastro.2012.06.031 22728514

[13] Rothschild D, Weissbrod O, Barkan E, Kurilshikov A, Korem T, Zeevi D, et al. Environment dominates over host genetics in shaping human gut microbiota. Nature 2018; 555: 210–5. doi: 10.1038/nature25973 29489753

[14] Yatsunenko T, Rey FE, Manary MJ, Trehan I, Dominguez-Bello MG, Contreras M, et al. Human gut microbiome viewed across age and geography. Nature 2012; 486: 222–7. doi: 10.1038/nature11053 22699611 PMC3376388

[15] Goodrich JK, Waters JL, Poole AC, Sutter JL, Koren O, Blekhman R, et al. Human genetics shape the gut microbiome. Cell 2014; 159: 789–99. doi: 10.1016/j.cell.2014.09.053 25417156 PMC4255478

[16] Graf D, Di Cagno R, Fåk F, Flint HJ, Nyman M, Saarela M, et al. Contribution of diet to the composition of the human gut microbiota. Microb Ecol Health Dis 2015; 26: 26164. doi: 10.3402/mehd.v26.26164 25656825 PMC4318938

[17] Wu GD, Chen J, Hoffmann C, Bittinger K, Chen YY, Keilbaugh SA, et al. Linking long-term dietary patterns with gut microbial enterotypes. Science (New York, N.Y.) 2011; 334: 105–8. doi: 10.1126/science.1208344 PMC3368382 21885731

[18] Valdes AM, Walter J, Segal E, Spector TD. Role of the gut microbiota in nutrition and health. BMJ (Clinical research ed.) 2018; 361: k2179. doi: 10.1136/bmj.k2179 PMC6000740 29899036

[19] Flint HJ, Duncan SH, Scott KP, Louis P. Links between diet, gut microbiota composition and gut metabolism. Proc Nutr Soc 2015; 74: 13–22. doi: 10.1017/S0029665114001463 25268552

[20] Koh A, De Vadder F, Kovatcheva-Datchary P, Bäckhed F. From dietary fiber to host physiology: short-chain fatty acids as key bacterial metabolites. Cell 2016; 165: 1332–45. doi: 10.1016/j.cell.2016.05.041 27259147

[21] Miyamoto J, Kasubuchi M, Nakajima A, Irie J, Itoh H, Kimura I. The role of short-chain fatty acid on blood pressure regulation. Curr Opin Nephrol Hypertens 2016; 25: 379–83. doi: 10.1097/mnh.0000000000000246 27490782

[22] Schoeler M, Caesar R. Dietary lipids, gut microbiota and lipid metabolism. Rev Endocr Metab Disord 2019; 20: 461–72. doi: 10.1007/s11154-019-09512-0 31707624 PMC6938793

[23] Candido FG, Valente FX, Grzeskowiak LM, Moreira APB, Rocha D, Alfenas RCG. Impact of dietary fat on gut microbiota and low-grade systemic inflammation: mechanisms and clinical implications on obesity. Int J Food Sci Nutr 2017, 69, 1–19. doi: 10.1080/09637486.2017.1343286 28675945

[24] Caesar R, Tremaroli V, Kovatcheva-Datchary P, Cani PD, Backhed F. Crosstalk between gut microbiota and dietary lipids aggravates WAT inflammation through TLR signaling. Cell Metab 2015; 22: 658–68. doi: 10.1016/j.cmet.2015.07.026 26321659 PMC4598654

[25] Cao W, Liu F, Li RW, Chin Y, Wang Y, Xue C, et al. Docosahexaenoic acid-rich fish oil prevented insulin resistance by modulating gut microbiome and promoting colonic peptide YY expression in diet-induced obesity mice. Food Sci Hum Wellness 2022; 11: 177–88. doi: 10.1016/j.fshw.2021.07.018

[26] Falony G, Joossens M, Vieira-Silva S, Wang J, Darzi Y, Faust K, et al. Population-level analysis of gut microbiome variation. Science (New York, N.Y.) 2016; 352: 560–4. doi: 10.1126/science.aad3503 27126039

[27] Zhernakova A, Kurilshikov A, Bonder MJ, Tigchelaar EF, Schirmer M, Vatanen T, et al. Population-based metagenomics analysis reveals markers for gut microbiome composition and diversity. Science (New York, N.Y.) 2016; 352: 565–9. doi: 10.1126/science.aad3369 PMC5240844 27126040

[28] Partula V, Mondot S, Torres MJ, Kesse-Guyot E, Deschasaux M, Assmann K, et al. Associations between usual diet and gut microbiota composition: results from the Milieu Intérieur cross-sectional study. Am J Clin Nutr 2019; 109: 1472–83. doi: 10.1093/ajcn/nqz029 31051503

[29] Noh H, Jang H-H, Kim G, Zouiouich S, Cho S-Y, Kim H-J, et al. Taxonomic composition and diversity of the gut microbiota in relation to habitual dietary intake in Korean adults. Nutrients 2021; 13: 366. doi: 10.3390/nu13020366 33530330 PMC7912254

[30] Trefflich I, Jabakhanji A, Menzel J, Blaut M, Michalsen A, Lampen A, et al. Is a vegan or a vegetarian diet associated with the microbiota composition in the gut? Results of a new cross-sectional study and systematic review. Crit Rev Food Sci Nutr 2020; 60: 2990–3004. doi: 10.1080/10408398.2019.1676697 31631671

[31] De Filippis F, Pellegrini N, Vannini L, Jeffery IB, La Storia A, Laghi L, et al. High-level adherence to a Mediterranean diet beneficially impacts the gut microbiota and associated metabolome. Gut 2016; 65: 1812–21. doi: 10.1136/gutjnl-2015-309957 26416813

[32] Asnicar F, Berry SE, Valdes AM, Nguyen LH, Piccinno G, Drew DA, et al. Microbiome connections with host metabolism and habitual diet from 1,098 deeply phenotyped individuals. Nat Med 2021; 27(2): 321–332. doi: 10.1038/s41591-020-01183-8 33432175 PMC8353542

[33] Ma W, Nguyen LH, Song M, Wang DD, Franzosa EA, Cao Y, et al. Dietary fiber intake, the gut microbiome, and chronic systemic inflammation in a cohort of adult men. Genome Med 2021; 13: 102. doi: 10.1186/s13073-021-00921-y 34140026 PMC8212460

[34] Companys J, Gosalbes MJ, Pla-Pagà L, Calderón-Pérez L, Llauradó E, Pedret A, et al. Gut microbiota profile and its association with clinical variables and dietary intake in overweight/obese and lean subjects: a cross-sectional study. Nutrients 2021; 13: 2032. doi: 10.3390/nu13062032 34199239 PMC8231825

[35] Li Y, Wang DD, Satija A, Ivey KL, Li J, Wilkinson JE, et al. Plant-based diet index and metabolic risk in men: exploring the role of the gut microbiome. J Nutr 2021; 151: 2780–9. doi: 10.1093/jn/nxab175 34114015 PMC8417919

[36] Gaundal L, Myhrstad MCW, Leder L, Byfuglien MG, Gjøvaag T, Rud I, et al. Beneficial effect on serum cholesterol levels, but not glycaemic regulation, after replacing SFA with PUFA for 3 d: a randomised crossover trial. Br J Nutr 2020; 125, 1–11. doi: 10.1017/S0007114520003402 PMC7944393 32873354

[37] Carlsen MH, Lillegaard ITL, Karlsen A, Blomhoff R, Drevon CA, Andersen LF. Evaluation of energy and dietary intake estimates from a food frequency questionnaire using independent energy expenditure measurement and weighed food records. Nutr J 2010; 9: 37. doi: 10.1186/1475-2891-9-37 20843361 PMC2949781

[38] Olsen A, Egeberg R, Halkjær J, Christensen J, Overvad K, Tjønneland A. Healthy aspects of the nordic diet are related to lower total mortality. J Nutr 2011; 141: 639–44. doi: 10.3945/jn.110.131375 21346102

[39] Roswall N, Sandin S, Löf M, Skeie G, Olsen A, Adami H-O, et al. Adherence to the healthy Nordic food index and total and cause-specific mortality among Swedish women. Eur J Epidemiol 2015; 30: 509–17. doi: 10.1007/s10654-015-0021-x 25784368

[40] Puaschitz NG, Assmus J, Strand E, Karlsson T, Vinknes KJ, Lysne V, et al. Adherence to the Healthy Nordic Food Index and the incidence of acute myocardial infarction and mortality among patients with stable angina pectoris. J Hum Nutr Dietet 2019; 32: 86–97. doi: 10.1111/jhn.12592 30091209

[41] Garnweidner-Holme L, Torheim LE, Henriksen L, Borgen I, Holmelid S, Lukasse M. Adherence to the Norwegian dietary recommendations in a multi-ethnic pregnant population prior to being diagnosed with gestational diabetes mellitus. Food Sci Nutr 2020; 8: 3031–40. doi: 10.1002/fsn3.1248 32724567 PMC7382101

[42] Totland THM, Kjerpeseth B, Lundberg-Hallén N, Helland-Kigen KM, Lund-Blix NA, Myhre JB, et al. Norkost 3 En landsomfattende kostholdsundersøkelse blant menn og kvinner i Norge i alderen 18-70 år, 2010-11. Oslo. Helsedirektoratet; 2012.

[43] NNR. Nordic nutrition recommendations 2012: integrating nutrition and physical activity; 9289326700. Copenhagen: Nordic Council of Ministers; 2014.

[44] Casén C, Vebø HC, Sekelja M, Hegge FT, Karlsson MK, Ciemniejewska E, et al. Deviations in human gut microbiota: a novel diagnostic test for determining dysbiosis in patients with IBS or IBD. Aliment Pharmacol Ther 2015; 42: 71–83. doi: 10.1111/apt.13236 25973666 PMC5029765

[45] Nasjonalt råd for ernæring. Kostråd for å fremme folkehelsen og forebygge kroniske sykdommer: Metodologi og vitenskapelig kunnskapsgrunnlag. Oslo, Helsedirektoratet, 2011.

[46] Hollister EB, Gao C, Versalovic J. Compositional and functional features of the gastrointestinal microbiome and their effects on human health. Gastroenterology 2014; 146: 1449–58. doi: 10.1053/j.gastro.2014.01.052 24486050 PMC4181834

[47] Jandhyala SM, Talukdar R, Subramanyam C, Vuyyuru H, Sasikala M, Nageshwar Reddy D. Role of the normal gut microbiota. World J Gastroenterol 2015; 21: 8787–803. doi: 10.3748/wjg.v21.i29.8787 26269668 PMC4528021

[48] Sorbara MT, Pamer EG. Microbiome-based therapeutics. Nat Rev Microbiol 2022, 20, 365–380. doi: 10.1038/s41579-021-00667-9 34992261

[49] Fisher CK, Mehta P. Identifying keystone species in the human gut microbiome from metagenomic timeseries using sparse linear regression. PLoS One 2014; 9: e102451. doi: 10.1371/journal.pone.0102451 25054627 PMC4108331

[50] Strömbeck A, Lasson A, Strid H, Sundin J, Stotzer P-O, Simrén M, et al. Fecal microbiota composition is linked to the postoperative disease course in patients with Crohn’s disease. BMC Gastroenterol 2020; 20: 130. doi: 10.1186/s12876-020-01281-4 32366222 PMC7197162

[51] Nomura K, Ishikawa D, Okahara K, Ito S, Haga K, Takahashi M, et al. Bacteroidetes species are correlated with disease activity in ulcerative colitis. J Clin Med 2021; 10: 1749. doi: 10.3390/jcm10081749 33920646 PMC8073534

[52] Agans R, Gordon A, Kramer DL, Perez-Burillo S, Rufián-Henares JA, Paliy O, et al. Dietary fatty acids sustain the growth of the human gut microbiota. Appl Environ Microbiol 2018; 84: e01525-18. doi: 10.1128/AEM.01525-18 30242004 PMC6193386

[53] David LA, Maurice CF, Carmody RN, Gootenberg DB, Button JE, Wolfe BE, et al. Diet rapidly and reproducibly alters the human gut microbiome. Nature 2014; 505: 559–63. doi: 10.1038/nature12820 24336217 PMC3957428

[54] Parker BJ, Wearsch PA, Veloo ACM, Rodriguez-Palacios A. The genus alistipes: gut bacteria with emerging implications to inflammation, cancer, and mental health. Front Immunol 2020; 11: 906. doi: 10.3389/fimmu.2020.00906 32582143 PMC7296073

[55] Kong Y, Li Y, Dai Z-R, Qin M, Fan H-L, Hao J-G, et al. Glycosaminoglycan from Ostrea rivularis attenuates hyperlipidemia and regulates gut microbiota in high-cholesterol diet-fed zebrafish. Food Sci Nutr 2021; 9: 5198–210. doi: 10.1002/fsn3.2492 34532028 PMC8441474

[56] Choi Y, Kwon Y, Kim D-K, Jeon J, Jang SC, Wang T, et al. Gut microbe-derived extracellular vesicles induce insulin resistance, thereby impairing glucose metabolism in skeletal muscle. Sci Rep 2015; 5: 15878. doi: 10.1038/srep15878 26510393 PMC4625370

[57] Jeong M-Y, Jang H-M, Kim D-H. High-fat diet causes psychiatric disorders in mice by increasing Proteobacteria population. Neurosci Lett 2019; 698: 51–7. doi: 10.1016/j.neulet.2019.01.006 30615977

[58] Hildebrandt MA, Hoffmann C, Sherrill-Mix SA, Keilbaugh SA, Hamady M, Chen YY, et al. High-fat diet determines the composition of the murine gut microbiome independently of obesity. Gastroenterology 2009; 137: 1716–24.e1–2. doi: 10.1053/j.gastro.2009.08.042 19706296 PMC2770164

[59] Rizzatti G, Lopetuso LR, Gibiino G, Binda C, Gasbarrini A. Proteobacteria: a common factor in human diseases. BioMed Res Int 2017; 2017: 9351507. doi: 10.1155/2017/9351507 29230419 PMC5688358

[60] Peters BA, Shapiro JA, Church TR, Miller G, Trinh-Shevrin C, Yuen E, et al. A taxonomic signature of obesity in a large study of American adults. Sci Rep 2018; 8: 9749. doi: 10.1038/s41598-018-28126-1 29950689 PMC6021409

[61] Xiao S, Fei N, Pang X, Shen J, Wang L, Zhang B, et al. A gut microbiota-targeted dietary intervention for amelioration of chronic inflammation underlying metabolic syndrome. FEMS Microbiol Ecol 2014; 87: 357–67. doi: 10.1111/1574-6941.12228 24117923 PMC4255291

[62] Sotos M, Nadal I, Marti A, Martínez A, Martin-Matillas M, Campoy C, et al. Gut microbes and obesity in adolescents. Proceed Nutr Soc 2008; 67: E20. doi: 10.1017/S0029665108006290

[63] Fei N, Zhao L. An opportunistic pathogen isolated from the gut of an obese human causes obesity in germfree mice. ISME J 2013; 7: 880–4. doi: 10.1038/ismej.2012.153 23235292 PMC3603399

[64] Palmu J, Salosensaari A, Havulinna AS, Cheng S, Inouye M, Jain M, et al. Association between the gut microbiota and blood pressure in a population cohort of 6953 individuals. J Am Heart Assoc 2020; 9: e016641. doi: 10.1161/JAHA.120.016641 32691653 PMC7792269

[65] Wilck N, Matus MG, Kearney SM, Olesen SW, Forslund K, Bartolomaeus H, et al. Salt-responsive gut commensal modulates TH17 axis and disease. Nature 2017; 551: 585–9. doi: 10.1038/nature24628 29143823 PMC6070150

[66] Dong J-Y, Szeto IMY, Makinen K, Gao Q, Wang J, Qin L-Q, et al. Effect of probiotic fermented milk on blood pressure: a meta-analysis of randomised controlled trials. Br J Nutr 2013; 110: 1188–94. doi: 10.1017/S0007114513001712 23823502

[67] Khalesi S, Sun J, Buys N, Jayasinghe R. Effect of probiotics on blood pressure: a systematic review and meta-analysis of randomized, controlled trials. Hypertension 2014; 64: 897–903. doi: 10.1161/hypertensionaha.114.03469 25047574

[68] Li J, Zhao F, Wang Y, Chen J, Tao J, Tian G, et al. Gut microbiota dysbiosis contributes to the development of hypertension. Microbiome 2017; 5: 14. doi: 10.1186/s40168-016-0222-x 28143587 PMC5286796

[69] Fu J, Bonder MJ, Cenit MC, Tigchelaar EF, Maatman A, Dekens JA, et al. The gut microbiome contributes to a substantial proportion of the variation in blood lipids. Circ Res 2015; 117: 817–24. doi: 10.1161/circresaha.115.306807 26358192 PMC4596485

[70] Koren O, Spor A, Felin J, Fåk F, Stombaugh J, Tremaroli V, et al. Human oral, gut, and plaque microbiota in patients with atherosclerosis. Proc Natl Acad Sci 2011; 108: 4592–8. doi: 10.1073/pnas.1011383107 20937873 PMC3063583

[71] Liu Y, Song X, Zhou H, Zhou X, Xia Y, Dong X, et al. Gut microbiome associates with lipid-lowering effect of rosuvastatin in vivo. Front Microbiol 2018; 9: 530. doi: 10.3389/fmicb.2018.00530 29623075 PMC5874287

[72] Tindall AM, McLimans CJ, Petersen KS, Kris-Etherton PM, Lamendella R. Walnuts and vegetable oils containing oleic acid differentially affect the gut microbiota and associations with cardiovascular risk factors: follow-up of a randomized, controlled, feeding trial in adults at risk for cardiovascular disease. J Nutr 2020; 150: 806–17. doi: 10.1093/jn/nxz289 31848609 PMC7138683

[73] Vacca M, Celano G, Calabrese FM, Portincasa P, Gobbetti M, De Angelis M. The controversial role of human gut lachnospiraceae. Microorganisms 2020; 8: 573. doi: 10.3390/microorganisms8040573 PMC7232163 32326636

[74] Sorbara MT, Littmann ER, Fontana E, Moody TU, Kohout CE, Gjonbalaj M, et al. Functional and genomic variation between human-derived isolates of lachnospiraceae reveals inter- and intra-species diversity. Cell Host Microbe 2020; 28: 134–46.e134. doi: 10.1016/j.chom.2020.05.005 32492369 PMC7351604

[75] Zhang X, Shen D, Fang Z, Jie Z, Qiu X, Zhang C, et al. Human gut microbiota changes reveal the progression of glucose intolerance. PLoS One 2013; 8: e71108. doi: 10.1371/journal.pone.0071108 24013136 PMC3754967

[76] Chávez-Carbajal A, Nirmalkar K, Pérez-Lizaur A, Hernández-Quiroz F, Ramírez-Del-Alto S, García-Mena J, et al. Gut microbiota and predicted metabolic pathways in a sample of Mexican women affected by obesity and obesity plus metabolic syndrome. Int J Mol Sci 2019; 20: 438. doi: 10.3390/ijms20020438 PMC6358992 30669548

[77] Kelly TN, Bazzano LA, Ajami NJ, He H, Zhao J, Petrosino JF, et al. Gut microbiome associates with lifetime cardiovascular disease risk profile among Bogalusa heart study participants. Circ Res 2016; 119: 956–64. doi: 10.1161/CIRCRESAHA.116.309219 27507222 PMC5045790

[78] Enget Jensen TM, Braaten T, Jacobsen BK, Barnung RB, Olsen A, Skeie G. Adherence to the Healthy Nordic Food Index in the Norwegian Women and Cancer (NOWAC) cohort. Food Nutr Res 2018; 62. doi: 10.29219/fnr.v62.1339 PMC6139480 30237757

[79] Huttenhower C, Gevers D, Knight R, Abubucker S, Badger JH, Chinwalla AT, et al. Structure, function and diversity of the healthy human microbiome. Nature 2012; 486: 207–14. doi: 10.1038/nature11234 22699609 PMC3564958

[80] Mueller S, Saunier K, Hanisch C, Norin E, Alm L, Midtvedt T, et al. Differences in fecal microbiota in different European study populations in relation to age, gender, and country: a cross-sectional study. Appl Environ Microbiol 2006; 72: 1027–33. doi: 10.1128/aem.72.2.1027-1033.2006 16461645 PMC1392899

[81] Haro C, Rangel-Zúñiga OA, Alcalá-Díaz JF, Gómez-Delgado F, Pérez-Martínez P, Delgado-Lista J, et al. Intestinal microbiota is influenced by gender and body mass index. PLoS One 2016; 11: e0154090. doi: 10.1371/journal.pone.0154090 27228093 PMC4881937

