# Supplementary material for: Gut microbiota is associated with dietary intake and metabolic markers in healthy individuals
Source: Food Nutr Res. 2022 Jun 23;66:10.29219/fnr.v66.8580. doi: 10.29219/fnr.v66.8580 (PMC9250133; doi:10.29219/fnr.v66.8580)
Supplement: Gut microbiota is associated with dietary intake and metabolic markers in healthy individuals [file FNR-66-8580-s001.docx]

**Supplementary table 1.** Daily dietary intake (g/day) of food groups included in the Healthy Nordic Food index

|  | Total (*n* = 48) | Men (*n* = 12) | Female (*n* = 36) |
| --- | --- | --- | --- |
|  | Median (25 - 75^th^ percentile) | Median (25 - 75^th^ percentile) | Median (25 - 75^th^ percentile) |
| Fish ^1^ | 47.7 (30.6 - 72.5) | 49.0 (33.9 - 62.3) | 47.5 (30.6 - 85.9) |
| Cabbages^2^ | 41.6 (20.1 - 70.8) | 33.4 (22.8 - 72.0) | 43.8 (16.4 - 65.9) |
| Apple and pears | 45.7 (17.6 - 81.3) | 19.5 (13.7 - 38.5) | 48.4 (27.1 - 87.9) |
| Root vegetables^3^ | 33.6 (33.7 - 70.3) | 24.6 (14.1 - 34.6) | 57.8 (27.3 - 72.4) |
| Oatmeal | 48.7 (14.8 - 76.8) | 50.5 (8.6 - 84.4) | 48.7 (18.0 - 68.6) |
| Whole grain bread ^4^ | 90.0 (39.5 - 135.0) | 135.0 (73.3 - 236.3) | 67.5 (39.5 - 90.0) |

^1^ Cod, pollack, salmon, trout, herring, mackerel, and shellfish
^2^ Cabbage, cauliflower, broccoli, Chinese cabbage, and brussels sprouts
^3^ Carrot, rutabaga
^4^ Whole grain bread > 50% whole meal flour.

**Supplementary Table 2**. Description of the Healthy Diet Score

| Component (food group) | Dietary recommendation^1^ | Cut-off values | Score |
| --- | --- | --- | --- |
| Whole grain | 4 portions/day | < 35 g/day  35-69 g /day > 70 g/day | 0  5  10 |
| Vegetables | ≥ 2.5 portions/day | 0 g/day  1-250 g/day  > 250g/day | 0  5  10 |
| Fruits and berries | ≥ 2.5 portions/day | 0 g/day 1-250 g/day  > 250g/day | 0  5  10 |
| Milk (low fat) | 3 portions/day | ≤ 3 times/week  > 3 - 6 times/week  ≥ 7 times/week | 0  5  10 |
| Fish | 2-3 times / week | < 1 times/week 1 time/week ≥ 2 times/week | 0  5  10 |
| Beans and lentils | Estimated: 2 times/week | < 1 times/week 1 time/week ≥ 2 times/week | 0  5  10 |
| Vegetable oils | MUFA 10-20 E%  PUFA 5-10 E% | < 10 E% ≥ 10 E%  < 5 E% ≥ 5 E% | 0  10  0  10 |
| Red/processed meats | Max 500 g/week | ≥ 2 times/week  1 time/week  < 1 time/week | 0  5  10 |
| Salt | Max 6 g salt/day | > 6 g/day ≤ 6 g/day | 0  10 |
| Sugar | Max 10 E% | > 10 E% ≤ 10 E% | 0  10 |
| Saturated fat | Max 10 E% | > 10 E% ≤ 10 E% | 0  10 |

^1^ Based on the Nordic nutrition recommendations [45]. Total score: 120 points
E%, percentage of total energy intake; MUFA, monounsaturated fatty acids; PUFA, polyunsaturated fatty acids.

**Supplementary table 3.** Overview of the phyla and bacterial names of the 48 bacteria DNA probes used in the GA-map™ Dysbiosis Test

| **Phylum** | **Name** |
| --- | --- |
| Actinobacteria | *Actinobacteria* |
| Actinobacteria | *Actinomycetales* |
| Actinobacteria | *Bifidobacterium* spp. |
| Bacteroidetes | *Alistipes* |
| Bacteroidetes | *Alistipes onderdonkii* |
| Bacteroidetes | *Bacteroides fragilis* |
| Bacteroidetes | *Bacteroides pectinophilus* |
| Bacteroidetes | *Bacteroides* spp. |
| Bacteroidetes | *Bacteroides* spp. & *Prevotella* spp. |
| Bacteroidetes | *Bacteroides stercoris* |
| Bacteroidetes | *Bacteroides zoogleoformans* |
| Bacteroidetes | *Parabacteroides johnsonii* |
| Bacteroidetes | *Parabacteroides* spp. |
| Firmicutes | *Firmicutes* |
| Firmicutes | *Bacilli* |
| Firmicutes | *Catenibacterium mitsuokai* |
| Firmicutes | *Clostridia* |
| Firmicutes | *Clostridium methylpentosum* |
| Firmicutes | *Clostridium* sp. |
| Firmicutes | *Coprobacillus cateniformis* |
| Firmicutes | *Dialister invisus* |
| Firmicutes | *Dialister invisus* & *Megasphaera micronuciformis* |
| Firmicutes | *Dorea* spp. |
| Firmicutes | *Eubacterium biforme* |
| Firmicutes | *Eubacterium hallii* |
| Firmicutes | *Eubacterium rectale* |
| Firmicutes | *Eubacterium siraeum* |
| Firmicutes | *Faecalibacterium prausnitzii* |
| Firmicutes | *Lachnospiraceae* |
| Firmicutes | *Lactobacillus ruminis* & *Pediococcus acidilactici* |
| Firmicutes | *Lactobacillus* spp. |
| Firmicutes | *Lactobacillus* spp. 2 |
| Firmicutes | *Phascolarctobacterium* sp. |
| Firmicutes | *Ruminococcus albus* & *Ruminococcus bromii* |
| Firmicutes | *Ruminococcus gnavus* |
| Firmicutes | *Streptococcus agalactiae* & *Eubacterium rectale* |
| Firmicutes | *Streptococcus salivarius* ssp*. thermophilus* & *S. sanguinis* |
| Firmicutes | *Streptococcus salivarius* ssp. *thermophilus* |
| Firmicutes | *Streptococcus* spp. |
| Firmicutes | *Streptococcus* spp. 2 |
| Firmicutes | *Veillonella* spp. |
| Firmicutes/Tenericutes/Bacteroidetes species | *Firmicutes* (various) |
| Proteobacteria | *Proteobacteria* |
| Proteobacteria | *Acinetobacter junii* |
| Proteobacteria | *Enterobacteriaceae* |
| Proteobacteria | *Shigella* spp. & *Escherichia* spp. |
| Tenericutes | *Mycoplasma hominis* |
| Verrucomicrobia | *Akkermansia muciniphila* |
